# Case Report: Exostosin 1-associated membranous nephropathy and Guillain-Barré syndrome: a common autoimmune etiology?

**DOI:** 10.3389/fneph.2025.1667619

**Published:** 2025-10-02

**Authors:** Mariana León-Póo, Eva López-Melero, Amir Shabaka, Carmen Guerrero-Márquez, María Barcenilla-López, Clara Cases-Corona, Enrique Gruss, Deborah Roldán

**Affiliations:** ^1^ Nephrology Department, Hospital Universitario Fundación Alcorcón, Madrid, Spain; ^2^ Nephrology Department, Hospital Universitario La Paz, Madrid, Spain; ^3^ Pathology Department, Hospital Universitario Fundación Alcorcón, Madrid, Spain; ^4^ Neurology Department, Hospital Universitario de Fuenlabrada, Madrid, Spain

**Keywords:** nephrotic syndrome, demyelinating polyneuropathy, contactin, exostosin 1, immunology

## Abstract

Membranous nephropathy is one of the most common causes of nephrotic syndrome in adults and is caused by the deposition of immune complexes in the subepithelial space of the glomerular basement membranes. On the other hand, Guillain-Barré syndrome is a type of acute, potentially fatal polyneuropathy, which is generally associated with an infection that serves as the initial immunological event and triggers immune-mediated disruption of the axon and/or myelin. We present the case of a 70-year-old patient with concurrent membranous nephropathy and Guillain-Barré syndrome, with subepithelial deposits in the renal biopsy positive for Exostosin 1, and who reached complete renal remission after treatment of Guillain-Barré syndrome with plasmapheresis and systemic corticosteroids, suggesting a common autoimmune origin for both entities.

## 1 Introduction

Membranous nephropathy (MN) is a histological pattern that can occur within a broad spectrum of diseases and is characterized by the deposition of immune complexes on the subepithelial side of the glomerular basement membranes (GBM), leading to a reactive change of the membrane around the deposits (1). This damage clinically translates into the appearance of proteinuria, generally causing nephrotic syndrome (NS); of which MN is one of its most common causes in adults (2).

Traditionally, MN has been classified as either primary (≈70% of cases) or secondary (≈30%) (1). Primary MN is an organ-specific autoimmune disease targeting podocyte antigens and is not associated with any systemic condition. In contrast, secondary MN is linked to identifiable systemic causes such as autoimmune diseases, infections, malignancies, drugs, or toxins (2).

In recent years, at least 14 target antigens have been identified in up to 90% of MN cases, many of which correlate with distinct clinical phenotypes. This has led to a proposed shift in classification from a primary/secondary framework to one based on the underlying antigen, which may better guide etiological investigations (1). The most common antigens include the M-type phospholipase A2 receptor (PLA2R; 70%–80% of cases) and thrombospondin type-1 domain-containing 7A (THSD7A; 1%–5%). More recently, exostosin 1 (EXT1) and exostosin 2 (EXT2) have been identified as novel target antigens associated with MN secondary to autoimmune diseases (3).

We present a case of MN with EXT1-positive deposits on kidney biopsy, occurring concurrently with Guillain-Barré syndrome, which may point to a common autoimmune etiology for both diseases.

## 2 Case description

We present the case of a 70-year-old male, a former smoker, with benign prostatic hyperplasia, for which he had a permanent urinary catheter and was awaiting surgical treatment, and no prior history of kidney disease. He presented to the Emergency Department with progressive weakness in the lower limbs (LL), particularly in the right lower limb (RLL), with no involvement of the upper limbs (UL), sensory symptoms, or fecal incontinence (bladder sphincter was not assessable due to the urinary catheter). Symptoms had started eight days prior to consultation. He also reported severe low back pain for the previous two weeks, for which he had been taking non-steroidal anti-inflammatory drugs (NSAIDs) for seven days. Additionally, he had a recent mild respiratory tract infection without fever a week before admission, which resolved spontaneously.

On physical examination, the patient exhibited bilateral LL edema and neurological findings including motor deficit in both LL (more pronounced in the RLL), generalized hyporeflexia in both LL and UL, and limited adduction of the right eye.

Laboratory tests revealed a significant decline in kidney function, with a serum creatinine of 2.42 mg/dl (baseline creatinine was 1 mg/dl three months before), along with marked hypoalbuminemia (2.3 g/dl), 24-hour proteinuria of 25 g, hypercholesterolemia, non-anion gap metabolic acidosis and mild hyperkalemia. Platelets were normal (414000/µl). Brain CT was normal, lumbar puncture revealed no abnormalities. Viral respiratory infection tests (SARS-CoV-2, respiratory syncytial virus, and influenza) were all negative.

The patient was diagnosed with progressive paraparesis and right ophthalmoplegia, with differential diagnoses including polyneuropathy and spinal cord compression. From a renal perspective, acute kidney injury (AKI) was initially suspected to be secondary to acute interstitial nephritis (AIN), likely due to recent NSAID use. Empirical corticosteroid treatment for suspected AIN was initially proposed but delayed due to its contraindication during the acute phase of Guillain-Barré Syndrome (GBS), as advised by neurologists.

A spinal MRI showed no signs of myelopathy, and electromyography (EMG) was consistent with axonal motor neuropathy. The neurological diagnosis was confirmed as the Acute Motor Axonal Neuropathy variant of GBS, and treatment with intravenous immunoglobulin (IVIG) was initiated.

Further laboratory workup included serological tests for hepatitis B and C, HIV, and syphilis, all of which were negative. Serum and urine electrophoresis did not reveal an M-band, and autoimmune tests [anti-gangliosides, ANA, anti-DNA, anti-Sm, anti-PLA2R and anti-contactin 1 (anti-CNTN1) antibodies] were also negative. Serum complement levels were normal.

Over the following days, the patient’s neurological condition worsened, with greater involvement of both LL and progressive UL involvement, as well as the appearance of progressive bulbar symptoms including dysarthria, spontaneous horizontal nystagmus in the left eye, paresis in left eye adduction, and absence of soft palate elevation. The patient required admission to the Intensive Care Unit and plasmapheresis was indicated every 48 hours (receiving a total of 5 sessions). Consequently, renal biopsy had to be delayed.

**Figure 1:**
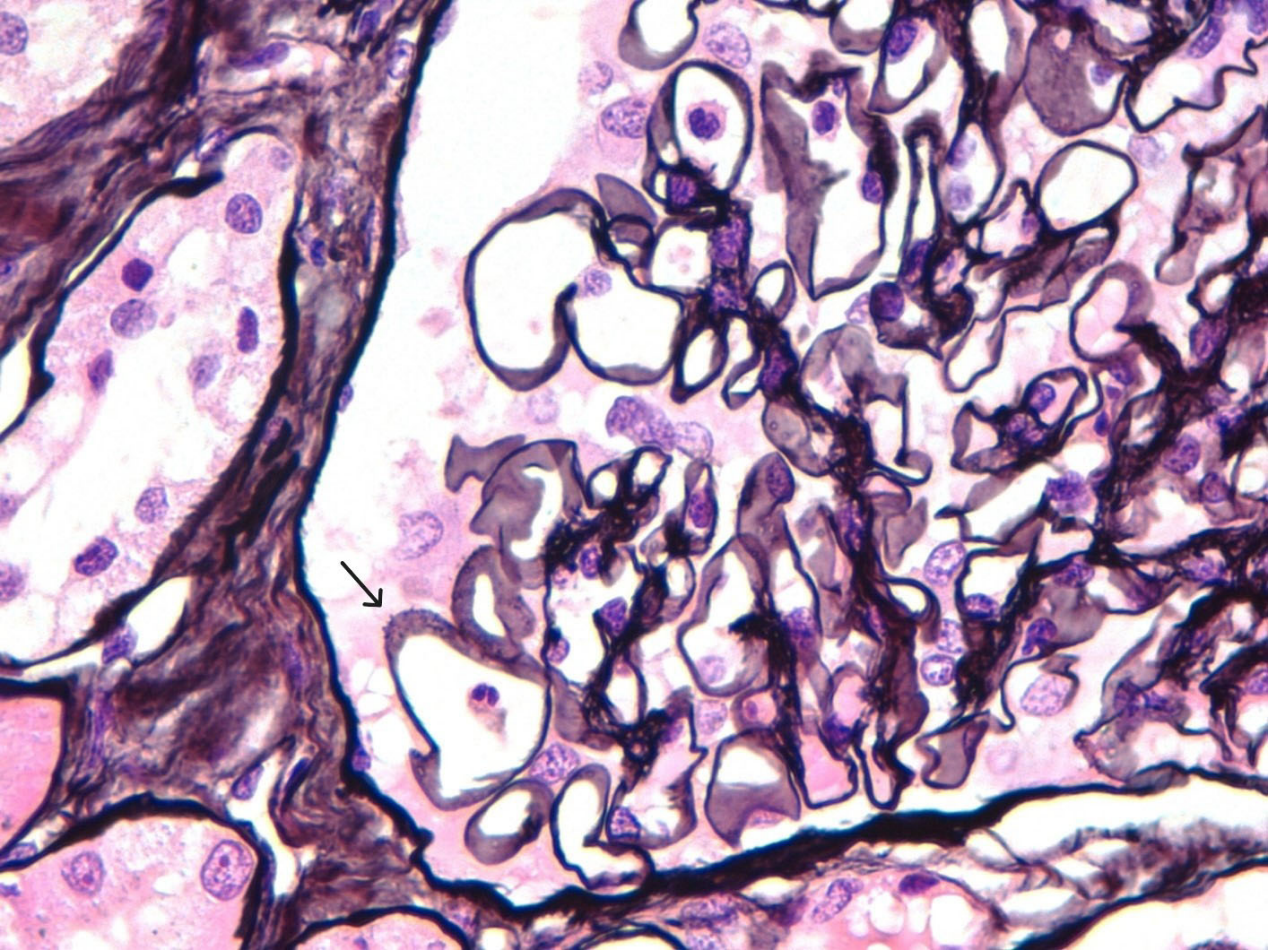
Silver staining revealing thickened GBM with evident spike formations.

**Figure 2:**
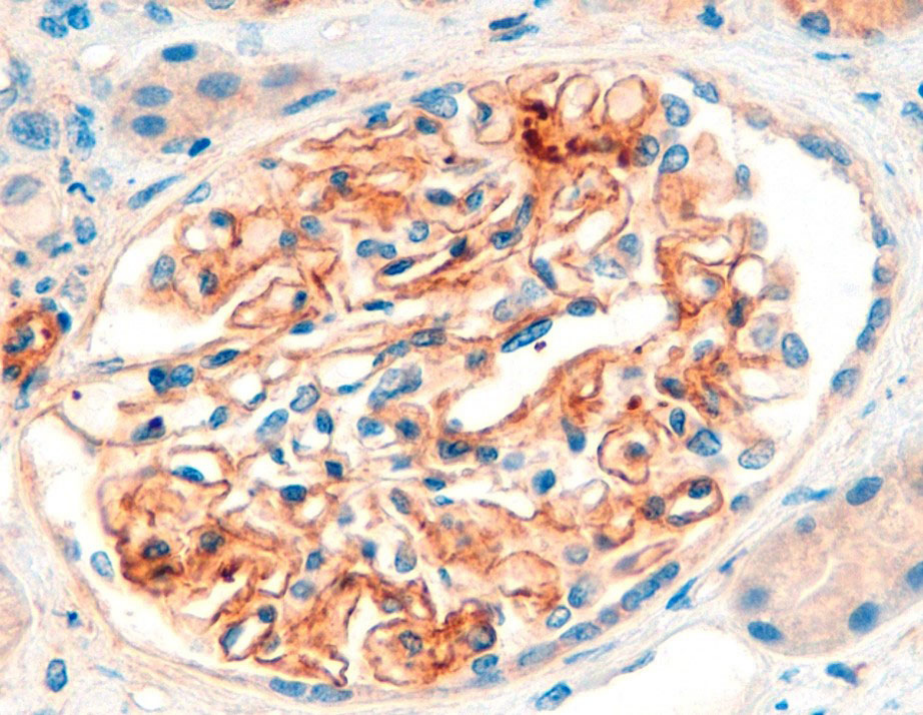
Immunohistochemistry (IHC) for EXT1 reveals intense granular deposits in the GBM.

After one week of hospitalization, both neurological and renal functions improved, allowing the initiation of steroid therapy with three boluses of 250 mg intravenous methylprednisolone, followed by 1 mg/kg/day, later switched to oral prednisone. Renal biopsy was performed, revealing 17 glomeruli with evident spike formations in the thickened GBM (**Figure 1**). On light microscopy, there were no signs of endocapillary hypercellularity or crescents. Silver staining showed evident spike formations in a thickened GBM (**Figure 1**). The interstitium showed moderate fibrosis with some atrophic tubules and sparse chronic inflammatory infiltrate. Direct immunofluorescence (DIF) revealed segmental subepithelial deposits positive for IgG, C3, kappa and lambda, and segmental subepithelial and mesangial deposits positive for IgM. DIF for IgA and C1q was negative. Immunohistochemistry (IHC) revealed intense granular deposits in the GBM positive for EXT1 (**Figure 2**), and negative for anti-PLA2R, THSD7A and EXT2. IgG4 staining was negative. Other subclasses of IgG were not available. Electron microscopy (EM) showed non-structured electron-dense subepithelial deposits in the GBM, with a spiculated reaction of the lamina densa and extensive podocyte effacement. The histological diagnosis was Exostosin 1 (EXT1)-associated membranous nephropathy.

Cancer screening was performed with a thoraco-abdomino-pelvic CT scan, thyroid ultrasound and tumor markers, all of which were normal except for an elevated PSA. Prostate biopsy showed no signs of malignancy, and the patient refused further endoscopic evaluation.

**Figure 3:**
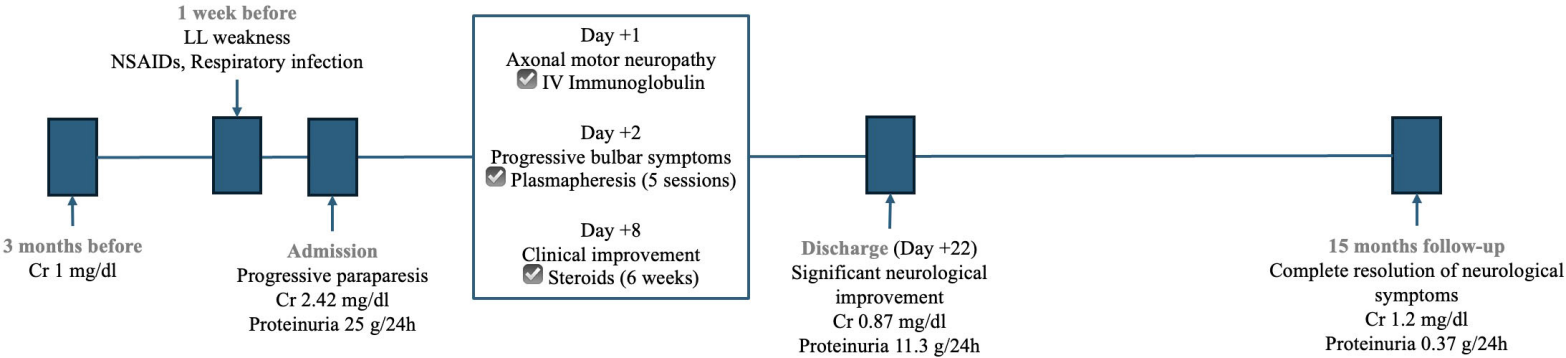
Timeline of kidney function, proteinuria and administered treatments.

After 22 days of hospitalization, the patient showed significant neurological improvement together with a progressive improvement of proteinuria and normalization of serum albumin and kidney function (**Figure 3**). He was discharged with a rapid tapering regimen of corticosteroids, completing 6 weeks of treatment. At 15-month follow-up, the patient had complete resolution of proteinuria and neurological symptoms. After 30 months, he remains in complete remission with no recurrence of symptoms.

## 3 Discussion

This is the first report of exostosin 1 (EXT1)-related membranous nephropathy (MN) that presented concurrently with Guillain-Barré Syndrome (GBS). A thorough review of the literature revealed that the association between MN and GBS has been recognized at least since 1973 (4, 5), with 12 published cases identified to date (**Table 1**) (4–14). Some of these cases suggest possible common etiological mechanisms, such as mercury poisoning (14) or Epstein-Barr virus (EBV) infection (12). However, the novelty of our case lies in the positivity for EXT1, prompting consideration of a potential common autoimmune origin.

**Table 1:** Literature review of case reports of GBS-associated membranous nephropathy.

Author	Year	Age (years)	Sex	SCr (mg/dl)	Proteinuria (g/day)	GBS variant	MN stage	Endo-/extracapillary proliferation	Basement membrane deposits	IgG subtype	Serum PLA2r	Tissue PLA2r	EXT1/2	Treatment	Outcome	Observations
Behan PO et al. (5)	1973	50	Male	1.1	13.5	ND	ND	No	IgG (3+), IgM (1+), α2D-globulin (3+)	IgG4 positive	ND	ND	ND	Prednisone, 6-mercaptopurine	No response	
Peters DK et al. (4)	1973	19	Male	N/A	3	ND	ND	ND	Ig, C3	ND	ND	ND	ND	Prednisone	Remission	
Talamo TS et al. (6)	1982	63	Male	“Normal”	3.7	ND	I	No	IgG (2+), IgM, C3	ND	ND	ND	ND	Prednisone	Remission	
Murphy BS et al. (7)	1986	69	Male	1.24	8	ND	II	No	IgG, C3	ND	ND	ND	ND	Not specified	Death	
		52	Male	1.02	7.9	ND	III	No	ND	ND	ND	ND	ND	Prednisolone	Death	
Nicholson GD et al. (8)	1989	49	Male	1.14	6.8	ND	II	No	ND	ND	ND	ND	ND	Prednisolone, Azathioprine	No response	Neurological improvement, no renal response
Kohli A et al. (9)	1992	18	Male	N/A	2.2	CIDP	II	No	ND	ND	ND	ND	ND	Prednisone	No response	Neurological improvement, no renal response
Panjwani M et al. (10)	1996	55	Male	0.8	15	ND	II	No	IgG, IgM	ND	ND	ND	ND	Prednisone	No response	
Emsley HC et al. (11)	2002	66	Male	3.1	2.59	AMAN	I	ND	ND	ND	ND	ND	ND	ACE inhibitors	Partial remission (GBS and MN)	
Meyer P et al. (12)	2010	12	Female	“Normal”	1.06	AIDP	I	No	IgG, C3	ND	ND	ND	ND	IV Immunoglobulins/ACE inhibitors, steroids and cyclosporine	Remission	EBV infection
Filippone EJ et al. (13)	2013	69	Male	1.4	20.14	AMAN	ND	ND	IgG	IgG4 negative	ND	ND	ND	IV Immunoglobulins	Death	
Yawei C et al. (14)	2021	44	Female	0.63	7.2	ND	ND	ND	IgG, C3, C1q	IgG4 predominant, IgG1, IgG2	Negative	ND	ND	IV Immunoglobulins, steroids/Succimer, steroids and tacrolimus	Complete remission GBS/Partial remission MN	Mercury intoxication. 1 year gap between GBS and MN
León-Póo et al.	2025	70	Male	2.42	25	AIDP	II	No	IgG, C3, Kappa, Lambda	IgG4 negative	Negative	Negative	Positive	Plasmapheresis, IV Immunoglobulins, Steroids	Remission	

AIDP, Acute Inflammatory Demyelinating Polyneuropathy; AMAN, Acute Motor Axonal Neuropathy; CIDP, Chronic Inflammatory Demyelinating Polyneuropathy; EXT1/2, Exostosin ½; GBS, Guillain-Barré Syndrome; ND, Not determined; PLA2r, Phospholipase A2 receptor; SCr, Serum creatinine.

GBS is the most common cause of flaccid paralysis worldwide. Seventy-six percent of affected patients report a preceding respiratory or gastrointestinal infection, with *Campylobacter jejuni* being the most frequently implicated pathogen. However, other microorganisms such as EBV, HBV, Influenza A, SARS-CoV-2, and Cytomegalovirus have also been associated with GBS. Additional triggers include immune checkpoint inhibitors and certain vaccines. The pathophysiology of the disease is characterized by two stages: an initial immunological trigger followed by an immune-mediated disruption of axons and/or myelin. This mechanism is well defined in *C. jejuni*-associated GBS, where molecular mimicry occurs between the lipo-oligosaccharides on its surface and gangliosides on peripheral nerve fibers, leading to production of cross-reactive antibodies against gangliosides, causing axoglial damage (15). In our patient’s case, the microorganism responsible for the respiratory infection was not identified, as the patient was asymptomatic upon admission, and anti-ganglioside antibodies were negative.

MN occurs due to the deposition of immune complexes on the subepithelial side of the GBM. In recent years, multiple antigens involved in immune complex formation have been identified (16), many of which are associated with specific clinical patterns. There is ongoing exploration into the use of these antigens for targeted treatments.

In 2019, a relationship between MN and EXT1/EXT2 was established (3). Exostosin (1 to 5) is a transmembrane protein located in the endoplasmic reticulum, involved in heparan sulfate synthesis. EXT1/EXT2 is positive in 7% of MN cases and is associated with the presence of autoimmune diseases such as systemic lupus erythematosus (SLE) and other connective tissue diseases (1). Moreover, accumulating evidence indicates that EXT1/EXT2-positive membranous lupus nephritis generally follows a more favorable clinical course compared with EXT1/EXT2-negative cases (17). However, since EXT1/EXT2-associated MN has only recently been described, further reports are needed to confirm these observations beyond lupus nephritis and to better delineate the clinical characteristics and prognosis of affected patients. To date, no circulating anti-EXT1/EXT2 antibodies have been identified. However, the coexistence of EXT1-positive MN with an immune-mediated neuropathy such as GBS suggests the possibility of convergent autoimmune pathways. In particular, molecular mimicry and aberrant antibody responses may represent a common trigger leading to parallel injury of peripheral nerves and the glomerular basement membrane.

Histologically, EXT1/2-related MN can present with features traditionally associated with secondary MN, including mesangial and even endocapillary proliferation, with positivity in DIF for not only IgG but also C1q, C3, IgA, or IgM. In EM, in addition to subepithelial deposits, mesangial and subendothelial deposits can be found, and sometimes tubule-reticular inclusions may be observed in cases associated with SLE. Immunohistochemistry for EXT1 and EXT2 typically shows positivity for both proteins, although EXT1 staining is usually more intense (16). In our case, the renal biopsy only showed EXT1 positivity, with no EXT2 staining. Direct immunofluorescence revealed granular subepithelial deposits of IgG, segmental granular deposits of IgM in both the subepithelium and mesangium, and deposits of C3, kappa, and lambda, similar to IgG. The patient had no clinical or serological evidence of systemic lupus erythematosus. The autoimmune panel was negative for ANA, anti-Jo1, anti-Scl70, anti-DNA, anti-SS-A, anti-SS-B, anti-RNP, and anti-Sm.

Anti-CNTN1 antibodies were identified in 2021 (18), and are responsible for 1% of MN cases (1). They have been associated with chronic inflammatory demyelinating polyneuropathy. Since our patient presented with acute axonal motor polyneuropathy, these antibodies were assessed but were negative.

This case report has some limitations, including the impossibility of establishing a definite causal relationship between GBS and EXT1-positive membranous nephropathy. Nevertheless, the strength of this report lies in being the first to describe the coexistence of these two conditions, raising the possibility of a shared autoimmune mechanism. As such, it provides valuable insights and may generate hypotheses for future research.

In conclusion, given the parallel course of both neurological and renal involvement, along with EXT1 positivity in the renal biopsy, we hypothesize that both GBS and MN are linked by a shared autoimmune mechanism, potentially mediated by a common antibody that remains to be identified.

## Data Availability

The original contributions presented in the study are included in the article/supplementary material. Further inquiries can be directed to the corresponding author.
